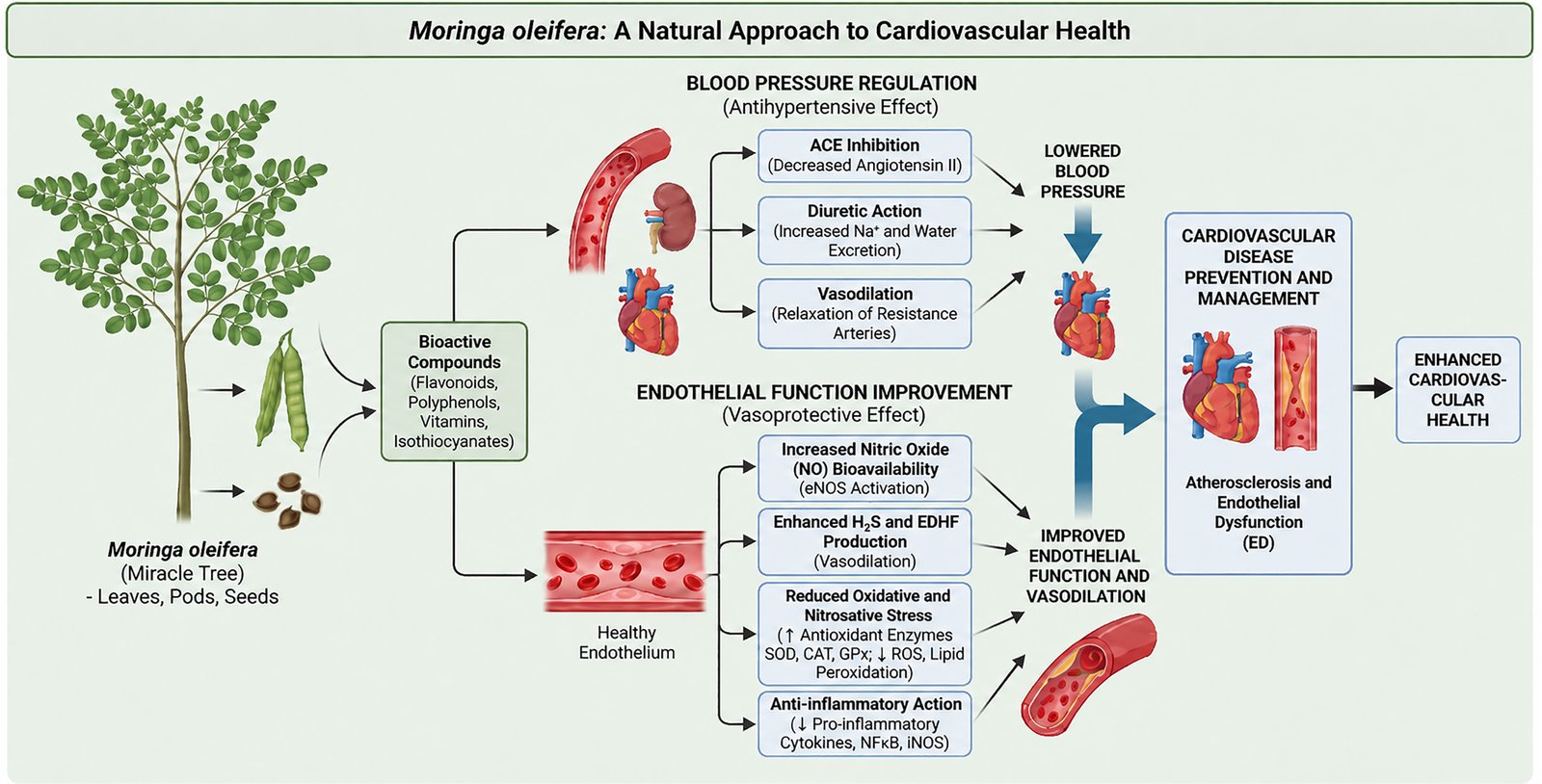# *Moringa oleifera* for blood pressure regulation and endothelial dysfunction—Mechanisms and therapeutic potential in cardiovascular disease: A review

**DOI:** 10.17305/bb.2026.13950

**Published:** 2026-06-03

**Authors:** Hemaniswarri Dewi Dewadas, Maheshen Mahindran, Teow Xin Yi, Sasmithaasree Sugumaran, Theophanie Victoria Sanjeevi Pandit, Sakshi Kumaresan, Seetha Munisamy, Muhammad Zulfiqah Sadikan

**Affiliations:** 1 Centre for Biomedical and Nutrition Research (CBNR), Faculty of Science, Universiti Tunku Abdul Rahman, Kampar, Perak, Malaysia; 2 Faculty of Medicine, Manipal University College Malaysia (MUCM), Bukit Baru, Malacca, Malaysia; 3 Faculty of Pharmacy and Health Sciences, Universiti Kuala Lumpur Royal College of Medicine Perak, Ipoh, Perak, Malaysia

**Keywords:** Atherosclerosis, cardiovascular disease, CVD, endothelial dysfunction, ED, *Moringa oleifera*, nitric oxide, NO

## Abstract

Cardiovascular diseases (CVDs) remain the leading cause of mortality worldwide. Atherosclerosis, the primary pathological mechanism underlying CVDs, culminates in endothelial dysfunction (ED). Reversible ED, characterized by impaired vascular relaxation and increased inflammation, represents a critical therapeutic target. *Moringa oleifera (M. oleifera)*, often referred to as the “miracle tree,” is garnering significant interest due to its broad spectrum of medicinal properties, including antihypertensive and endothelial-protective activities. Rich in bioactive compounds such as flavonoids, polyphenols, and vitamins, this plant exhibits diverse pharmacological activities.This review aims to explore the role of *M. oleifera* in hypertension management and the improvement of endothelial function. Clinical evidence primarily supports the blood pressure-lowering effects of *M. oleifera*. Preclinical studies, however, suggest additional mechanisms, including enhanced nitric oxide (NO) bioavailability, angiotensin-converting enzyme (ACE) inhibition, potent antioxidant activity, diuretic effects, and direct endothelial protective actions. Furthermore, preclinical findings elucidate that its vasodilatory and diuretic effects may involve NO-mediated pathways, hydrogen sulfide (H_2_S) signaling, and endothelium-derived hyperpolarizing factor (EDHF)-dependent mechanisms, thereby highlighting its potential therapeutic value in vascular health. Its antioxidant capacity may also confer protection against the oxidative and nitrosative stress commonly associated with hypertension- and diabetes-induced endothelial dysfunction. While promising, the majority of current evidence is derived from animal models and small-scale clinical studies. Therefore, further well-designed, large-scale human studies are imperative to definitively establish the efficacy and safety of *M. oleifera* as a natural therapeutic agent for cardiovascular health. This review underscores the critical need for additional research to fully clarify the therapeutic potential of *M. oleifera* in the prevention and management of CVDs.

## Introduction

Cardiovascular diseases (CVDs) represent a leading global health concern and a major cause of mortality worldwide. Global statistics indicate a significant rise in CVD mortality, increasing from 12.1 million in 1990 to 20.5 million cases in 2021. In Malaysia, CVD poses a substantial public health burden, with mortality rates escalating over the past four decades [1]. In 2016, CVD accounted for 13.2% of total deaths, making it the leading cause, followed by stroke at 6.9%. Furthermore, CVD contributed to 21.65% of mortality among the top ten leading causes of death in Malaysian hospitals in 2018. Atherosclerosis, characterized by arterial plaques composed of fats, cholesterol, calcium, and cellular waste, is the primary pathological driver of CVD. The accumulation of these plaques narrows arteries, impeding oxygen supply to vital organs and leading to myocardial infarction when coronary arteries are affected [2].

Endothelial dysfunction (ED) is a primary causative factor in the pathogenesis of atherosclerosis. The endothelium, a monolayer of cells lining the innermost layer of blood vessels, functions as a critical barrier facilitating gas and metabolite exchange. It secretes molecules that modulate vascular relaxation and contraction, along with enzymes that regulate blood clotting, immunity, and platelet adhesion. CVD risk factors such as smoking, aging, hypercholesterolemia, hypertension, and hyperglycemia lead to endothelial cell damage, subsequently resulting in ED. ED is characterized by dysregulated vascular reactivity, marked by increased production of vasoconstrictors, reduced availability of vasodilators, diminished antithrombotic factors, and persistent inflammation [3]. In its early stages, ED is reversible, and many therapeutic strategies aim to restore endothelial function.

**Figure 1.**
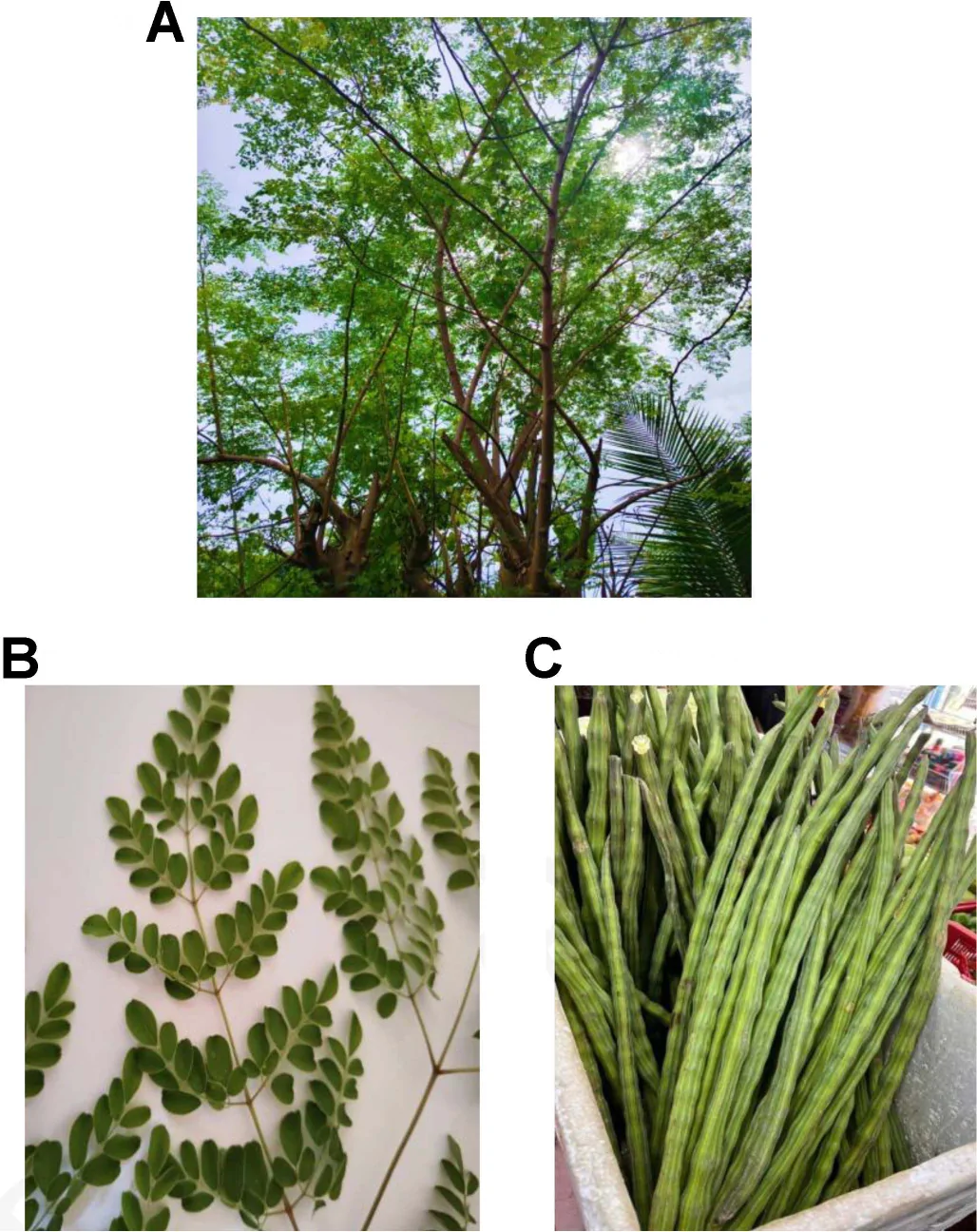
**Morphological features and major usable parts of *Moringa oleifera.*** (A) Whole *M. oleifera* tree showing its characteristic branching habit. (B) Representative leaves, the plant part most commonly used in nutritional and medicinal preparations. (C) Mature pods, another edible and traditionally used plant part discussed in relation to the phytochemical and therapeutic potential of *M. oleifera*. Abbreviation: *M. oleifera*, *Moringa oleifera*.

Given the critical role of ED in the development and progression of hypertension and atherosclerotic cardiovascular disease [3], increasing attention has focused on therapeutic strategies capable of improving vascular function and reducing cardiovascular risk. In recent years, natural products and plant-derived bioactive compounds have garnered significant scientific interest due to their antioxidant, anti-inflammatory, vasodilatory, and cardioprotective properties. Among these, *Moringa oleifera* (*M. oleifera*) has emerged as a promising medicinal plant with potential antihypertensive and endothelial protective effects [4–6]. Previous studies suggest that *M. oleifera* may contribute to blood pressure (BP) regulation through multiple mechanisms, including antioxidant activity [5, 6], modulation of nitric oxide bioavailability [5], angiotensin-converting enzyme (ACE) inhibition [5], and vascular relaxation pathways associated with endothelial function [4–6].

## *M. oleifera* as a medicinal plant

*M. oleifera*, often referred to as the “miracle tree,” is highly valued for its extensive medicinal and nutritional attributes. As the sole genus in the Moringaceae family, *Moringa* comprises 13 species, with *M. oleifera* being the most widely cultivated. Traditionally used as a food ingredient, it has gained popularity as a food supplement due to its high nutritional content. Nearly all parts of this versatile plant are utilized in treating various diseases [7, 8]. The leaves, seeds, and pods of *M. oleifera* are particularly valuable, with the leaves being the most frequently utilized (Figure 1). *M. oleifera* extracts are rich in diverse bioactive compounds, including vitamin C, vitamin E, polyphenols, carotenoids, flavonoids, phenolic acids, alkaloids, lycopene, isothiocyanates, glucosinolates, saponins, and tannins. These compounds collectively exhibit a wide array of pharmacological effects, such as anti-inflammatory, anticancer, neuroprotective, and hepatoprotective properties. They are also reported to reduce blood sugar, enhance fertility, alleviate pain, treat depression, normalize thyroid function, induce diuresis, and lower blood lipid levels [9].

### Positioning of the current review and literature gap

Recent reviews have highlighted the potential of *M. oleifera* as a natural therapeutic agent for cardiovascular health. Menichetti et al. (2025) evaluated both animal and human studies, reporting that the strongest evidence for BP reduction is associated with *M. oleifera* leaves, although conclusions from clinical studies remain limited by the small number of well-designed randomized controlled trials [4]. Qadi et al. (2025) focused on the antihypertensive mechanisms of *M. oleifera*, including ACE inhibition, nitric oxide-mediated vasodilation, antioxidant and anti-inflammatory activities, diuretic effects, and lipid regulation [5]. Similarly, Sharma and Banerjee (2025) analyzed evidence on hypertension-related outcomes, emphasizing the effects of *M. oleifera* on BP, oxidative stress, inflammation, endothelial function, and other cardiometabolic parameters [6]. Collectively, these reviews support the therapeutic potential of *M. oleifera* in CVD prevention and management.

Despite these promising findings, several important limitations persist within the current body of evidence. While a number of clinical and observational studies have reported reductions in systolic blood pressure (SBP) and diastolic blood pressure (DBP) following *M. oleifera* supplementation [10–17], the findings are not entirely consistent and warrant cautious interpretation. Significant variation exists in study populations, baseline health conditions, supplement formulations, extraction methods, dosage regimens, intervention duration, and outcome measurements, making direct comparisons across studies challenging. Recent systematic reviews and meta-analyses have similarly highlighted substantial heterogeneity in study quality, methodological approaches, and outcome reporting, thereby limiting the translation of current findings into clinical practice [4–6].

Moreover, antihypertensive effects have not been uniformly observed across all populations. While several studies reported significant reductions in SBP and DBP, others demonstrated only modest or statistically nonsignificant changes, particularly in patients with metabolic syndrome [18]. Short-term studies often show acute BP reductions [12, 15], whereas longer-duration interventions generally report more modest but sustained improvements [16, 17]. Nevertheless, many available studies are limited by small sample sizes, inadequate randomization, insufficient control of confounding variables, and short follow-up periods, thereby weakening causal interpretations.

Another significant limitation across both primary studies and recent reviews is the restricted direct assessment of endothelial function biomarkers in human studies. Although BP reductions are frequently reported, the mechanistic relationship between *M. oleifera* supplementation and improved endothelial function remains largely inferential rather than directly validated clinically. Current mechanistic interpretations are predominantly extrapolated from preclinical evidence involving nitric oxide bioavailability, ACE inhibition, antioxidant activity, hydrogen sulfide signaling, and endothelial-related vascular pathways.

Additionally, while *M. oleifera* is generally considered safe when consumed as a food source, evidence regarding the long-term safety of medicinal preparations and concentrated extracts remains limited. Existing reviews suggest that the safety profile of *M. oleifera* may vary depending on the plant part used, extraction method, dosage, duration of exposure, and phytochemical composition [19]. Variability among commercial preparations may further contribute to differences in therapeutic efficacy and safety outcomes, emphasizing the need for product standardization and quality control. Furthermore, comprehensive toxicological evaluations, long-term clinical safety data, and assessments of potential herb–drug interactions remain scarce [8, 19].

**Table 1:** Preclinical evidence demonstrating the hypotensive effects of *M. oleifera*

**Study**	**Parts of *M. oleifera* and animal model**	**Experimental design**	**Dose**	**Blood pressure outcome**
[25]	Almost all the parts (leaves, twigs, stems, roots, pods and seeds) Animal: Spontaneously hypertensive (SH) and normotensive (NT).	Rats were randomized into 12 groups of five rats each from SH and NT.	Both SH and NT rats were fed 1000 mg/kg/day (aqueous and ethanolic extracts) for 14 days	Significant reduction in SBP and DBP in SH rats, except aqueous stem extract. No significant effects were observed in NT rats, suggesting that *M. oleifera* might not cause hypotension in individuals with normal BP.
[26]	Leaf Animal: Preeclampsia induced diabetes in Wistar rats.	30 pregnant rats divided into 6 groups: two control groups and the other 4 groups.	100, 200, 400, and 800 mg/kg/day.	Significant reduction in SBP was observed in rats receiving 400 mg/kg/day of *M. oleifera*. No significant changes were seen in rats receiving low doses of *M. oleifera.*
[27]	Leaf Animal: L-NAME induced preeclampsia Wistar rats	Rats randomized into six groups (n = 4): 1. Control group (no treatment received) 2. L-NAME with 50 mg of ethanolic extract of *M. oleifera* leaves (EMOL) 3. L-NAME with 100 mg of EMOL 4. L-NAME with 200 mg of EMOL 5. L-NAME with low dose of 1.35 mg aspirin.	50, 100, and 200 mg/kg/day. BP was measured on gestation days 0, 11, and 19.	Both EMOL (50, 100, and 200 mg/kg/day) and low-dose aspirin prevented a significant increase in SBP and DBP in preeclamptic rats, showing that EMOL has a similar effect to low-dose aspirin in preventing the increase in blood pressure.
[28]	Leaf Animal: L-NAME induced hypertensive female ICR strain mice	Mice randomised into 7 groups with 5 mice in each group. Group 1: Control group receiving distilled water Group 2: Vehicle group Group 3: Captopril-treated group (20 mg/kg/day) Groups 4 and 5: Methanol extract-treated groups (0.3 and 0.01 g/kg/day, respectivel y) Groups 6 and 7: Ethyl acetate extract-treated groups (0.3 and 0.01 g/kg/day, respectively)	Mice treated with methanol (ME) or ethyl acetate (EA) extracts of *M. oleifera* at 0.01 and 0.3 g/kg/day for 25 days.	Significant decrease in BP compared to the hypertensive group. Both ME and EA extracts lowered SBP in L-NAME-induced hypertensive mice
[29]	Leaf Animal: L-NAME induced hypertensive Male Wistar rats	Rats were divided into 6 groups Group 1: Normal control rats administered only distilled water (DW) Group 2: Normal rats administered MOE (60 mg/kg) Group 3: L-NAME hypertensive rats administered DW Group 4: L-NAME treated rats administered Cap (5 mg/kg) Group 5: L-NAME treated rats administered *M. oleifera* (30 mg/kg) Group 6: L-NAME treated rats administered *M. oleifera* (60 mg/kg)	30 and 60 mg/kg/day of *M. oleifera* or positive control, Captopril (5 mg/kg) for 3 weeks.	SBP decreased in a dose-dependent manner: 189 mmHg to 165 mmHg at 30 mg/kg, and 189 mmHg to 148 mmHg at 60 mg/kg; this effect is comparable to that of captopril.

**Abbreviations:** BP, blood pressure; DBP, diastolic blood pressure; DW, distilled water; EA, ethyl acetate; EMOL, ethanolic extract of *Moringa oleifera* leaves; ICR, Institute of Cancer Research; L-NAME, Nω-nitro-L-arginine methyl ester; ME, methanol extract; MOE, *Moringa oleifera* extract; NT, normotensive; SBP, systolic blood pressure; SH, spontaneously hypertensive.

Therefore, this review critically evaluates the role of *M. oleifera* in BP regulation and endothelial function by integrating clinical findings with mechanistic evidence related to endothelial regulation. Furthermore, this review highlights current knowledge gaps, methodological limitations, safety considerations, and future research priorities to clearly define the therapeutic potential of *M. oleifera* in cardiovascular disease prevention and management.

### Clinical evidence on *M. oleifera* in BP regulation and endothelial function

Nitric oxide (NO), a crucial signaling molecule in the cardiovascular system, plays a pivotal role in vascular homeostasis. It acts as a potent vasodilator, regulating BP and ensuring endothelial integrity. Impaired NO synthesis is commonly observed in hypertension and diabetes, conditions associated with ED and CVD [20]. *M. oleifera* has been documented for its antihypertensive activity, positioning it as a potential therapeutic agent for hypertension and atherosclerosis. Its endothelial function-enhancing and BP-lowering effects have been extensively studied in various animal and human trials. Numerous clinical studies highlight the significant potential of *M. oleifera* as an alternative method for hypertensive treatment. For example, in healthy volunteers, *M. oleifera* leaf consumption led to a reduction in BP, particularly among individuals with high salt intake [10]. However, the clinical implications of BP reduction in normotensive individuals remain unclear, requiring further studies to determine if these effects are transient, physiologically adaptive, or potentially associated with adverse outcomes. Similarly, Sailesh et al. reported reductions in SBP and DBP in stage-1 hypertensive patients not receiving antihypertensive medication [11]. A study by Arun et al. demonstrated that daily intake of *M. oleifera* leaf tea for 15 days resulted in a decrease in BP across patients with varying severities of hypertension, suggesting sustained antihypertensive effects even in individuals with pre-existing hypertension [12].

The impact of *M. oleifera* extends beyond systemic BP reduction, encompassing a notable decrease in intraocular pressure (IOP) [13]. Administration of *M. oleifera* leaves in healthy individuals has consistently demonstrated significant, dose-dependent reductions in both BP and IOP, with higher doses yielding more pronounced effects.

Further research corroborates these findings, showing that increasing doses of aqueous *M. oleifera* extracts correlate with greater reductions in BP and IOP in normotensive subjects [14]. Oral administration of these extracts produced significant, dose-dependent reductions in both parameters. A recent study also highlighted *M. oleifera* supplementation’s dual benefits, not only reducing BP but also improving blood glucose levels in patients with type 2 diabetes [15]. Similarly, diabetic patients receiving *M. oleifera* supplementation experienced dose-dependent reductions in SBP, fasting blood glucose, and lipid parameters [16]. These observations suggest that *M. oleifera* may offer additional metabolic and cardiovascular advantages for individuals with diabetes and hypertension. Furthermore, *M. oleifera* supplementation has been linked to significant reductions in BP and body weight, particularly among obese hypertensive individuals [17]. While most studies indicate a significant hypotensive effect of *M. oleifera*, it is noteworthy that some Mauritian communities hold a perception that consuming *M. oleifera* leaves and pods increases BP [21–22]. In contrast, a recent double-blind, randomized, placebo-controlled trial involving 73 patients with metabolic syndrome reported that *M. oleifera* supplementation improved several metabolic and inflammatory markers, including reductions in glycated hemoglobin (HbA1c), insulin, high-sensitivity C-reactive protein (hs-CRP), and malondialdehyde (MDA) levels. Although reductions in SBP and DBP were also observed, these changes did not achieve statistical significance [18].

Recent high-level evidence further contextualizes these findings. A 2026 systematic review and meta-analysis by Mokgalaboni K. et al. reported an association between *M. oleifera* intake and reductions in both SBP and DBP. However, substantial heterogeneity in study design, dosing strategies, and population characteristics limits definitive clinical interpretation [23]. Similarly, other recent systematic reviews report modest or variable effects, underscoring the necessity for larger, well-designed clinical trials [24]. Collectively, these findings suggest a general trend toward antihypertensive effects, though variability persists due to differences in study design, dosage, and population demographics. The antihypertensive effects of *M. oleifera* in animal studies are summarized in Table 1.

### Mechanisms of antihypertensive action (preclinical evidence)

*M. oleifera*’*s* antihypertensive effects are attributed to several postulated mechanisms, as illustrated in Figure 2. These include ACE inhibition, diuretic action, and increased NO production. An experimental study by Acuram and Chichioco-Hernandez [28] demonstrated that *M. oleifera* may reduce BP through ACE inhibitory activity. Ethyl acetate extracts exhibited significant ACE inhibition, primarily due to kaempferol-3-O-glucoside and quercetin-3-O-glucoside, with efficacy comparable to captopril. *In vivo* findings further showed that methanol (ME) and ethyl acetate (EA) extracts of *M. oleifera* leaves, administered at doses of 0.01 and 0.3 g/kg/day for 25 days, significantly reduced BP in Nω-nitro-L-arginine methyl ester (L-NAME)-induced hypertensive mice.

**Figure 2.**
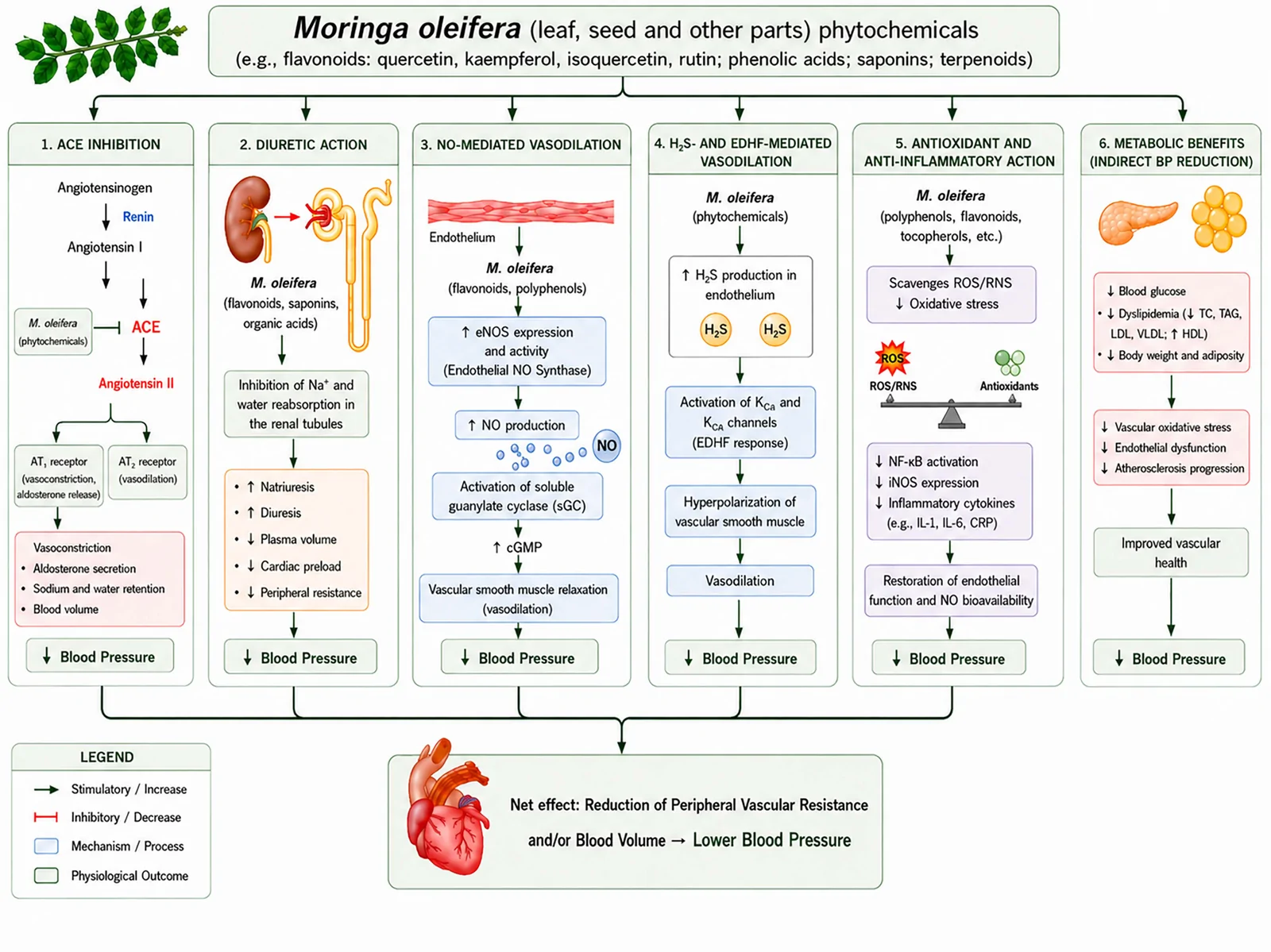
**Proposed mechanisms underlying the antihypertensive and endothelial-protective effects of Moringa oleifera.** Bioactive phytochemicals from *M. oleifera*, including flavonoids, phenolic acids, saponins, and terpenoids, may lower blood pressure through multiple complementary pathways. These include inhibition of ACE and attenuation of angiotensin II–mediated vasoconstriction; diuretic activity through reduced renal sodium and water reabsorption; NO-mediated vasodilation through increased eNOS activity, NO production, and sGC–cGMP signaling; and H_2_S- and EDHF-mediated vascular relaxation through activation of K_ATP_ and K_Ca_ channels. Additional antioxidant and anti-inflammatory effects may reduce ROS/RNS generation, NF-κB activation, iNOS expression, and inflammatory cytokine signaling, thereby supporting restoration of endothelial function and NO bioavailability. Metabolic benefits, including improved glycemic control, lipid profile, body weight, and vascular oxidative stress, may further contribute indirectly to blood pressure reduction and vascular protection. Collectively, these mechanisms decrease peripheral vascular resistance and/or blood volume, resulting in lower blood pressure. **Abbreviations:** ACE, angiotensin-converting enzyme; AT_1_, angiotensin II type 1 receptor; AT_2_, angiotensin II type 2 receptor; BP, blood pressure; cGMP, cyclic guanosine monophosphate; CRP, C-reactive protein; EDHF, endothelium-derived hyperpolarizing factor; eNOS, endothelial nitric oxide synthase; H_2_S, hydrogen sulfide; HDL, high-density lipoprotein; IL-1, interleukin-1; IL-6, interleukin-6; iNOS, inducible nitric oxide synthase; K_ATP, ATP-sensitive potassium channel; K_Ca, calcium-activated potassium channel; LDL, low-density lipoprotein; *M. oleifera*, *Moringa oleifera*; NF-κB, nuclear factor kappa B; NO, nitric oxide; ROS/RNS, reactive oxygen species/reactive nitrogen species; sGC, soluble guanylate cyclase; TAG, triacylglycerol; TC, total cholesterol; VLDL, very-low-density lipoprotein.

These findings suggest that *M. oleifera* extracts may modulate multiple pathways associated with hypertension, including ACE inhibition and NO-related mechanisms. Similar antihypertensive effects mediated through ACE inhibition have been reported in other preclinical studies [25]. In this particular study, various plant parts, including leaves, twigs, stems, roots, pods, and seeds, were evaluated. Spontaneously hypertensive (SH) and normotensive (NT) rats were administered ethanolic and aqueous *M. oleifera* extracts at 1000 mg/kg each for 14 days. All SH rats exhibited significant reductions in SBP and DBP, with the exception of those receiving the aqueous stem extract. No significant effect was observed in NT rats, suggesting that *M. oleifera* may not induce hypotension in normotensive subjects. The observed ACE inhibition and BP reduction were attributed to the phytochemical composition of *M. oleifera*, including chlorogenic acid, β-sitosterol, stigmasterol, campesterol, and kaempferol-3-O-rutinoside. The greatest ACE inhibition, exceeding 50%, was recorded at 40 and 80 µg/mL concentrations in ethanolic leaf extract, aqueous leaf extract, and ethanolic pod extract, indicating potential hypotensive activity. Furthermore, the ethanolic extract demonstrated significant diuretic activity, with urine excretion in SH rats comparable to that in hydrochlorothiazide-treated rats. This diuretic activity can be ascribed to flavonoids, saponins, and organic acids present in *M. oleifera*, which may inhibit the reabsorption of water and ions in the renal tubules.

The antihypertensive activity of *M. oleifera* is pharmacologically integrated with its diuretic activity. Both the whole fraction of *M. oleifera* and its gum have demonstrated diuretic properties [30]. Diuretic activity is generally associated with antihypertensive effects because diuretics promote the excretion of salt and water in urine, thereby decreasing fluid volume in the circulatory system and consequently reducing BP. Some research indicates that the diuretic properties of *M. oleifera* are comparable to those of furosemide [31] and thiazide [32]. Tahkur et al. further demonstrated that *M. oleifera* leaf extract exhibits dose-dependent diuretic activity, which is greater than that of the control but less effective than hydrochlorothiazide [33]. Overall, ACE inhibition and diuretic activity appear to act synergistically in contributing to the observed antihypertensive effects.

In addition to ACE inhibition and diuretic activity, *M. oleifera* also promotes endothelium relaxation due to the presence of vasodilators. A study by Aekthammarat et al. found that *M. oleifera* enhances endothelial nitric oxide synthase (eNOS) synthesis, leading to the relaxation of resistance arteries and a consequent reduction in arterial BP [34]. In this study, intravenous administration of *M. oleifera* aqueous leaf extract (1-30 mg/kg) to male Wistar rats decreased mean arterial pressure (MAP) in a dose-dependent manner and caused relaxation of mesenteric arterial beds. This effect was attributed to the enhancement of eNOS observed in primary human pulmonary artery endothelial cells. The study also confirmed that *M. oleifera* (3-30 µg/mL) stimulates NO production in human pulmonary artery endothelial cells, even when previously suppressed by L-NAME, a known eNOS inhibitor. This highlights *M. oleifera*’s role in restoring NO levels, a critical factor in combating hypertension and maintaining a healthy vascular system. The impact of *M. oleifera* on NO synthase activity was also examined in diabetic models. In a study by Sierra-Campos et al., diabetic rats treated with *M. oleifera* leaf extract at a dosage of 200 mg/kg for 21 days showed a reversal of compromised NO levels, increased inducible nitric oxide synthase (iNOS) activity, and decreased paraoxonase 1 (PON1) activity observed in untreated diabetic rats. PON1, a calcium-dependent antioxidant esterase, plays a significant role in endothelial health. *M. oleifera* leaf extract treatment in this study reversed oxidative damage, preserved PON1 activity, and normalized nitric oxide synthase (NOS) levels, demonstrating a protective role against diabetes-induced endothelial dysfunction [35].

*M. oleifera*’s vascular protective effects in aging Wistar rats suggest its ability to combat oxidative stress (OS) and promote endothelial function, which typically declines with age. A study by Randriamboavonjy et al. revealed that *M. oleifera* seeds ameliorate aging-related ED in middle-aged Wistar rats [36]. In this study, 46-week-old rats were administered 750 mg/kg/day *M. oleifera* seed powder for 4 weeks, with 16-week-old rats serving as controls. Endothelial function of the aorta and mesenteric arteries was assessed using isometric contraction. The results indicated that *M. oleifera* seed treatment improved endothelial function in middle-aged Wistar rats by enhancing eNOS activity in the aorta, suggesting that aortic endothelial function is largely mediated by NO. *M. oleifera* seeds were also found to enhance endothelium-dependent hyperpolarizing factor (EDHF)-dependent relaxation of mesenteric arteries. While NO primarily accounts for endothelium-dependent relaxation of blood vessels, in some small arteries, endothelium-dependent relaxation is mediated by EDHF. EDHF is an important mediator of endothelium-dependent relaxation in small resistant arteries where NO plays a less significant role. In a study by Aekthammarat et al., L-NAME was injected into Wistar rats to induce hypertension, and approximately 100 mg of *M. oleifera* leaf extract, containing isoquercetin, catechin, gallic acid, tannic acid, quercetin, rutin, and apigenin, was administered to the hypertensive rats [37]. Results demonstrated that *M. oleifera* induces endothelial relaxation in the mesenteric artery mediated by EDHF-dependent and endothelium-independent mechanisms. The endothelium-independent effect is linked to the blockade of extracellular Ca^2+^ entry through voltage-operated and receptor-operated Ca^2+^ channels, as well as the blockade of sarcolemmal Ca^2+^ mobilization through inositol trisphosphate receptor Ca^2+^ channels. These findings suggest that *M. oleifera* may act as a natural vasodilator and a potential adjunctive therapy in hypertension.

*M. oleifera* not only enhances NO generation but also releases hydrogen sulfide (H_2_S), both contributing to endothelium-dependent relaxation. Aekthammarat et al. demonstrated that *M. oleifera* leaf extract stimulates H_2_S production in spontaneously hypertensive rat mesenteric resistance arteries. While other vasodilators, such as NO and prostanoids, are inhibited by incubating mesenteric vessels with L-NAME (an eNOS synthase inhibitor) and indomethacin (a cyclooxygenase inhibitor), respectively, endothelium-dependent relaxation persisted. This relaxation was abolished by DL-propargylglycine, an inhibitor of the enzyme responsible for H_2_S generation. This suggests that H_2_S likely mediates endothelium-dependent relaxation, involving the activation of adenosine triphosphate-sensitive potassium channels (K_ATP_) and calcium-activated potassium channels (K_Ca_) channels and myoendothelial gap junctional communication [38].

### *M. oleifera* in oxidative nitrosative pathway modulation

The ability of *M. oleifera* to inhibit OS is considered a crucial factor in preventing hypertension and ED. A study by Aekthammarat et al. evaluated the antioxidant activity of *M. oleifera*. L-NAME hypertensive rats exhibited elevated vascular O_2_ synthesis and increased plasma and thoracic aorta MDA levels compared to normotensive controls. These hypertensive rats also showed a decline in antioxidant activities, reflected by reduced superoxide dismutase (SOD) and catalase (CAT) levels. Treatment of induced-hypertensive rats with *M. oleifera* (30 and 60 mg/kg/day) resulted in decreased vascular O_2_ production and MDA content in both plasma and the thoracic aorta. Furthermore, *M. oleifera* significantly enhanced SOD and CAT activities in L-NAME-treated rats. Beyond its antioxidant potential, *M. oleifera* treatment restored acetylcholine-induced relaxation, which was attenuated in L-NAME-treated rats. This study proposed that the antihypertensive effect of *M. oleifera* may stem from its capacity to induce vasorelaxation and mitigate vascular OS [29].

In addition to its vasoprotective effects, *M. oleifera* significantly reduced blood glucose levels, potentially slowing ED progression. In pregnant diabetic rats, *M. oleifera* leaf powder at 200 mg/kg/day lowered blood glucose, while a reduction in BP was observed at 400 mg/kg/day in diabetic rats [26]. Elevated blood glucose levels are known to trigger increased OS, leading to endothelial cell injury. This research indicates that *M. oleifera* acts as an antioxidant, inhibiting free radical production and restoring homeostatic balance. Consistent with previous findings, *M. oleifera* leaf extract (300 mg/kg body weight) also inhibited OS in diabetic rats [39]. While antioxidant activities of glutathione peroxidase (GPx), glutathione reductase (GRD), SOD, and CAT were reduced in diabetic rats, oral administration of *M. oleifera* significantly enhanced these activities in the heart tissue of diabetic rats. The antioxidant properties of *M. oleifera* may be attributed to compounds such as 2-benzenedicarboxylic acid, hexadecanoic acid, heptadecanoic acid, 9,12,15-octadecatrienal, and DL-alpha-tocopherol. Lipid peroxidation products, including conjugated dienes (CD), hydroperoxides (HP), and thiobarbituric acid-reactive substances (TBARS), were elevated in diabetic rats due to decreased antioxidant enzyme levels, contributing to free radical generation and lipid peroxidation. *M. oleifera* treatment significantly lowered these lipid peroxidation products and exhibited an antidiabetic effect by reducing glucose levels in diabetic-induced rats. No significant differences were observed in lipid peroxidation products, antioxidant enzymes, or glutathione levels between untreated normotensive rats and *M. oleifera*-treated rats.

Randriamboavonjy et al. [40] reported that spontaneously hypertensive rats exhibited increased oxidative and nitrosative vascular stress. This was evidenced by a reduction in circulating free 8-isoprostane levels, coupled with increased vascular p22phox and p47phox expressions, and superoxide dismutase 2 (SOD2) upregulation. Nitrite and C-reactive protein concentrations were also elevated in SHR. Treatment with *M. oleifera* seeds (750 mg) for 20 weeks not only decreased oxidative and nitrosative stress but also demonstrated anti-inflammatory action. Elevated OS levels correlated with inflammation and upregulation of iNOS via nuclear factor kappa B (NF-κB) p65v transcription factor activity, which decreased following *M. oleifera* treatment. Functional experiments showed that carbachol-induced endothelium relaxation was enhanced in *M. oleifera*-treated resistance arteries due to its antioxidant effect.

The vasoprotective effect of *M. oleifera* was also observed in another group of rats that developed ED [41]. A high-fat diet (HFD) was used to induce vascular dysfunction and obesity in Wistar albino rats. *M. oleifera* leaf extract was administered at 200 mg/kg/day to one HFD-fed group and 400 mg/kg/day to another for 3 weeks. *M. oleifera* augmented acetylcholine-induced relaxation, which was blunted in the HFD group. The *M. oleifera*-treated group showed reductions in body weight, waist circumference, Lee index, body mass index (BMI), liver weight to body weight ratio (LW/BW), and retroperitoneal fat pad weight to body weight ratio (RFPW/BW) compared to HFD-treated rats. *M. oleifera* intake significantly improved the lipid profile, whereas the HFD group exhibited a substantial increase in total cholesterol, triglycerides, low-density lipoprotein (LDL), and very low-density lipoprotein (VLDL) levels, alongside a decrease in high-density lipoprotein (HDL) levels. Histopathological examination revealed that the aortas of HFD-fed rats were infiltrated with fat in the form of adipocytes, had compromised elastic fiber layers, and an increased collagen-to-elastic ratio due to a thicker collagen layer compared to control diet-fed rats. *M. oleifera* at 400 mg/kg/day resulted in an almost normal aortic architecture, including general features, collagen-to-elastic ratio, and elastic fiber condition. This study confirmed that *M. oleifera* restored vascular structural and functional integrity and ameliorated histopathological alterations associated with obesity. Similarly, another investigation by Othman et al. [42] demonstrated that *M. oleifera* leaf extract (600 mg) administered for 6 weeks to HFD-fed rats reduced body weight and improved glucose levels, lipid fractions, and metabolic hormones, reflecting an anti-obesity effect. *M. oleifera* was also found to reduce OS by acting as an antioxidant and improved liver function enzymes. The effects of *M. oleifera* were comparable to those of antihyperlipidemic drugs (simvastatin) or a combination of both drugs, with the combination being more effective than either drug alone.

### Future directions

Despite robust preclinical and preliminary clinical evidence supporting the antihypertensive and endothelium-protective properties of *M. oleifera*, several critical research gaps must be addressed before its integration into clinical practice. A significant portion of the current literature relies on animal models or small-scale, short-term human trials. While these studies offer valuable mechanistic insights, they inherently limit the external validity and generalizability of the findings. To definitively establish the efficacy, dose-response relationships, and long-term safety of *M. oleifera*, large-scale, randomized, double-blind, placebo-controlled clinical trials are imperative across diverse populations. These trials must account for inter-individual variability influenced by age, gender, comorbidities, dietary factors, and genetic polymorphisms that impact endothelial function and responses to oxidative stress. Only through such high-quality clinical investigations can the true translational potential of *M. oleifera* as a cardiovascular therapeutic be fully elucidated [43].

Furthermore, mechanistic research should extend beyond conventional pathways such as NO generation, ACE blockade, and diuretic activity. While these mechanisms are recognized, the multifactorial nature of hypertension and endothelial dysfunction suggests the involvement of additional molecular targets. Emerging evidence indicates that *M. oleifera* modulates H_2_S signaling pathways, EDHF, and oxidative-nitrosative stress; however, the comparative importance and interplay of these pathways remain poorly defined. Next-generation omics technologies, including transcriptomics, proteomics, and metabolomics, hold promise for identifying novel bioactive compounds and signaling cascades, thereby enhancing mechanistic understanding and potentially revealing synergistic interactions with conventional antihypertensive medications.

Another equally critical area of focus is standardization and formulation development. *M. oleifera* is a complex phytochemical matrix, and the heterogeneity of its bioactive composition, influenced by plant organ, extraction method, and cultivation conditions, limits reproducibility and hinders regulatory approval. The development of standardized extracts with consistent phytochemical profiles and high quality is essential to ensure reliable therapeutic effects [44]. Additionally, comprehensive pharmacokinetic and pharmacodynamic studies are urgently needed to understand the absorption, bioavailability, metabolism, and tissue distribution of its bioactive constituents [45]. Such insights will be crucial for establishing robust dosing regimens, mitigating potential drug-herb interactions, and optimizing efficacy when administered alongside existing cardiovascular therapies [46].

Finally, given the global burden of cardiovascular disease and the growing interest in plant-based therapeutics, there is a compelling need to integrate *M. oleifera* into broader preventive strategies. Beyond its antihypertensive effects, *M. oleifera* exhibits pleiotropic actions, including antioxidant, anti-inflammatory, and antidiabetic activities, each capable of independently reducing cardiovascular risk. Future research should adopt holistic study designs that evaluate composite outcomes such as vascular stiffness, endothelial progenitor cell function, and the incidence of major cardiovascular events, rather than focusing solely on isolated biomarkers. This approach will facilitate a more comprehensive assessment of *M. oleifera*’s therapeutic potential and may support its future role as a valuable adjunct or alternative to conventional pharmacotherapy for the prevention and management of cardiovascular disease [47].

## Conclusion

*M. oleifera* demonstrates significant potential for cardiovascular health due to its capacity to lower BP and enhance endothelial function, both critical for the prevention and control of cardiovascular diseases. While initial preclinical studies have shown promising data regarding its vascular protective and antihypertensive effects, most findings are currently confined to laboratory settings and animal models. To firmly validate these observations and fully realize the therapeutic value of *M. oleifera*, larger human studies and rigorous clinical trials are imperative. These future investigations are essential to establish the efficacy, safety, and therapeutic potential of *M. oleifera* as a natural intervention for cardiovascular health.

## Supplemental data

Graphical abstract